# Shared Goals, Different Barriers: A Qualitative Study of UK Veterinarians' and Farmers' Beliefs About Antimicrobial Resistance and Stewardship

**DOI:** 10.3389/fvets.2019.00132

**Published:** 2019-04-25

**Authors:** Sarah E. Golding, Jane Ogden, Helen M. Higgins

**Affiliations:** ^1^Faculty of Health & Medical Sciences, School of Psychology, University of Surrey, Guildford, United Kingdom; ^2^Institute of Veterinary Science, University of Liverpool, Neston, United Kingdom

**Keywords:** antimicrobial resistance, prescribing, stewardship, veterinarians, farmers, beliefs, animal welfare, one health

## Abstract

Although much research has investigated the drivers of inappropriate antimicrobial prescribing in human medicine, equivalent research in veterinary medicine is in its infancy. This qualitative study used a critical incident approach to explore farm veterinarians' (vets) and farmers' beliefs about antimicrobial use and antimicrobial stewardship. Semi-structured interviews were conducted with 13 vets and 12 farmers in the UK, who worked mostly with beef cattle, dairy cattle and sheep, but a minority also worked with pigs or poultry. An inductive thematic analysis was conducted to explore how vets and farmers understood their responsibilities toward stewardship and antimicrobial resistance (AMR) and to identify key similarities and differences between the professions. The analysis generated four themes: “A shared conflict between ideals and behaviour,” “Barriers to stewardship: the vets' perspective,” “Barriers to stewardship: the farmers' perspective,” and “A shared ambivalence: ownership vs. other-blaming.” Vets and farmers demonstrated good understanding of stewardship but their treatment decisions are not always aligned to stewardship principles. Various barriers to improving antimicrobial stewardship were discussed by vets and farmers, but they placed differing emphasis on specific barriers. Faced with these barriers and an awareness that antimicrobial usage is not always aligned to stewardship principles, vets and farmers expressed frustration and a sense of ambivalence toward stewardship, and also engaged in other-blaming for the problem of AMR. In conclusion, vets and farmers in this study seem motivated to be antimicrobial stewards but feel challenged by the day-to-day reality of their jobs; they experience ambivalence toward their responsibilities for AMR, which may negatively impact their motivation to always act as antimicrobial stewards. Successfully tackling AMR will require change at the individual-, group-, and societal-level. Future interventions to improve antimicrobial usage in livestock farming could be situated within a social ecological framework, where other-blaming between professions is seen as a result of the interplay between psychological and contextual factors. Other-blaming could be reduced using a social identity approach; a common ingroup identity could be created by encouraging vets and farmers to focus on their common goal, namely a shared desire to promote animal welfare through optimal antimicrobial stewardship.

## Introduction

AMR is a truly global, cross-species problem, and solving the challenge requires a collaborative, One Health approach, taking account of human, animal, and environmental health (1–3). In human medicine, much research has explored doctors' beliefs regarding antimicrobial prescribing and stewardship (4), and various interventions to reduce inappropriate prescribing have been trialled and systematically reviewed [e.g., (5, 6)]. Both qualitative and quantitative research methods have been extensively used in this context and results indicate doctors' antimicrobial prescribing is influenced by clinical and non-clinical factors; furthermore, social science perspectives are recognized as key to understanding the psychological and contextual drivers of inappropriate antimicrobial use across human medicine (7–9). Psychological factors are conceptualized in this study as cognitive, affective, and interpersonal factors; contextual factors are those that are external to individuals, such as environmental conditions, economic circumstances, or resource availability, but which nonetheless impact on an individual's or group's ability to act (10). Context may influence an individual's perceptions, beliefs, and motivations, but an individual's psychology may also shape their context (10–12).

In contrast, equivalent research with farm animal veterinarians (vets) and farmers is in its infancy. A rapid evidence assessment of existing research into antimicrobial usage in livestock found much research has focused on establishing patterns of usage, with only limited research on vets' and farmers' treatment decisions (13). In some countries, such as the UK, even though antimicrobials are prescription-only veterinary medicines, farmers can, and frequently do, administer antimicrobials to animals they have diagnosed themselves, without the vet being present (14, 15). It is therefore important to consider both farmers' and vets' treatment decisions. Interventions aimed at changing vets' and farmers' treatment decisions are beginning to be developed (16–19), but given the limited research into treatment decisions, there are likely to be gaps in current understanding of the psychological and contextual drivers of those decisions in vets and farmers.

Of those studies that have explored treatment decisions, most used quantitative methodology (13). Surveys suggest that vets' treatment decisions are not just based on clinical factors such as presumed diagnosis or clinical history; decisions are also influenced by non-clinical psychological and contextual factors, such as perceived expectations of farmers or farm infrastructure (20–22). The extent to which vets report non-clinical factors as important does, however, vary (22). Farmers' treatment decisions are also influenced by non-clinical psychological and contextual factors, such as concerns about a lack of time or worries that produce yields might fall if antimicrobials are removed (15, 23). Furthermore, surveys suggest that psychological factors that influence treatment decisions and opportunities for change, such as beliefs and intentions, can vary between countries, possibly due to contextual factors such as legislative differences (24, 25).

Although surveys provide insight into what factors might influence treatment decisions, a greater understanding of why different factors influence decisions can be elicited by using qualitative methodology. Qualitative approaches to data collection allow participants to respond more freely and in greater depth about their thoughts surrounding their decision-making. To date, however, only a few qualitative studies have explored vets' and farmers' antimicrobial treatment decisions, and mostly just in specific, limited contexts. Much of this research has focused either on the pig industry, or on udder health in dairy cows. Focus groups and interviews with UK pig vets' found prescribing was influenced by various psychological and contextual factors, including pressure from farmers and economic issues (26, 27), whilst UK pig farmers also reported pressures related to production costs as potentially driving antimicrobial usage (26). Vets and farmers working with pigs or poultry from five European countries felt treatment decisions were influenced by economic factors and the nature of intensive farming systems (28). Amongst UK cattle vets, asked to consider antimicrobial therapy to prevent udder infections, varying risk perceptions influenced their willingness to change treatment protocols (29). Dairy farmers in The Netherlands and Germany also reported that their decisions to extend antimicrobial treatment for clinical mastitis were influenced by their beliefs about the risk of disease recurrence (30). Dutch farm vets expressed conflicting interests in their prescribing decisions, including risk avoidance, and a financial dependence on clients (31). Cost of production and risk management were also identified as influencing antimicrobial usage during interviews with UK vets and farmers from the pig, poultry, and dairy industries; this study, however, focused on investigating the broader context of social practices surrounding antimicrobial usage, rather than the psychological factors influencing treatment decisions (32).

Existing qualitative research therefore suggests that, at least in some contexts, economic concerns and risk perceptions may influence vets' and farmers' treatment decisions. What these psychological and contextual influences mean for vets' and farmers' antimicrobial stewardship decisions, however, remains unclear. Vets and farmers mostly demonstrate good knowledge about the need for responsible antimicrobial prescribing, but express some scepticism of the agricultural contribution to health risks, especially for human health (26–29, 32, 33). There is also evidence suggesting AMR is not always prioritized in prescribing decisions. For example, although AMR awareness amongst Italian cattle vets was high, over half reported using critically important antimicrobials as a first-line treatment for calf diarrhoea (34). Farmers also do not appear to prioritize AMR in their treatment decisions (35, 36) and may not be fully aware of the health risks from AMR (23, 28, 35).

To date, very little research into vets' and farmers' antimicrobial treatment decisions has been social science-led (32), but there is increasing recognition of social scientists' potential contribution to tackling AMR (37). Existing research suggests that, as with doctors, both psychological and contextual factors likely influence vets' and farmers' antimicrobial treatment decisions. There remains, however, a paucity of in-depth explorations of the beliefs that UK farm vets and farmers, working across a range of species, hold about their antimicrobial treatment decisions, especially in relation to stewardship principles. This study takes a psychosocial perspective, informed by a social ecological approach (11, 12), to explore the dynamic interplay between psychological (e.g., beliefs, risk perceptions) and contextual (e.g., economics, industry norms) factors. Social ecological frameworks have been utilized in other veterinary contexts to identify barriers to improved biosecurity on dairy farms (38). Furthermore, to the authors' knowledge, no studies have used a critical incident methodology (39) in this context. A critical incident approach can support the interview process as it asks interviewees to consider a specific event (the critical incident) in detail. By encouraging vets and farmers to consider actual treatment decisions, rather than treatment decisions in the abstract, a critical incident approach can elicit a richer dataset and additional insight into vets' and farmers' decisions. This study therefore aimed to address these gaps from a psychosocial perspective by applying a qualitative, critical incident approach to explore (1) farm vets' and farmers' beliefs about antimicrobial use on-farm (2) their beliefs about antimicrobial resistance and (3) how these beliefs may or may not support antimicrobial stewardship. The analysis was inductive and data-driven, but conclusions drawn are discussed in terms of a social ecological approach to health promotion (11, 12) that highlights the interplay between individual-level, group-level, and societal-level factors.

## Methods

### Participants, Recruitment, and Ethics

Participants were recruited opportunistically by distributing flyers at industry events, via the researchers' professional network, and through snowball sampling; participants were encouraged to advertise the study to their networks, and especially to those they felt may have differing views on the issues discussed. Of those approached by email, five people declined to participate (one due to time pressures; four did not respond to the invitation). No participant had any prior relationship with the interviewer, SG, although some were part of the extended professional network of one co-researcher, HH. Inclusion criteria were: vets working in private veterinary practice with livestock (any species, full- or part-time) and farmers keeping at least one species of livestock for commercial purposes. No restrictions were set based on demographic variables; a screening question ensured all participants were involved with antimicrobial treatment decisions on farms.

Recruitment continued until saturation was reached (40). There are no set guidelines for an appropriate sample size for qualitative interviews, but it is acknowledged there are diminishing returns from later interviews (41). Indeed, one systematic analysis of the coding process demonstrated that 92% of all codes, and 97% of high-frequency codes, were identified within the first 12 interviews (42).

Participation was voluntary, and participants were told they could withdraw, without providing a reason, at any time until a pre-specified date; all participants were offered the opportunity to enter a draw for a £25 shopping voucher. Participants provided written informed consent online, before their interview; on two occasions where consent was not completed online, SG took verbal consent before starting those interviews. A favourable ethical opinion for the study was granted by the University of Surrey's Ethics Committee. The study has been reported in line with the COREQ (consolidated criteria for reporting qualitative studies) checklist (43).

### Design

In-depth telephone interviews were conducted by SG between April and September 2016; telephone interviews enabled recruitment from across the UK. Participants were free to choose their location for the interviews but were requested to be somewhere they would not be disturbed. Interviews were audio-recorded, were guided using a semi-structured interview schedule (see Supplementary Material) and lasted between 27 and 54 min.

### The Interview

The development of the interview schedule was informed by critical incident methodology (39); the application of a “critical incident technique” to qualitative interviews has been demonstrated elsewhere (44, 45). The application of this technique here was dual-purpose: (1) to prompt participants to discuss concrete examples, to aid their thinking and (2) to elicit different examples of antimicrobial usage incidents. To avoid the risk that participants responded narrowly by focussing on a specific incident, they were asked about three different incidents and prompts were used to encourage them to elaborate. By encouraging participants to discuss different examples in this way, a richer dataset can be collected.

The schedule was piloted with two vets and two farmers to check acceptability and understanding of questions; some language was amended following feedback, but the broad structure and topic areas remained the same. Predominantly open questions were used, with follow-up prompts as needed, to facilitate free discussion. Participants were first asked about three antimicrobial prescribing (vets)/usage (farmers) incidents: “Please can you tell me about a recent example of when [you had to prescribe/use an antibiotic/the decision to prescribe/use an antibiotic was less clear-cut/you could have prescribed/used antibiotics but decided not to]?” Participants were then asked about the wider issue of AMR; questions included “what does the term antimicrobial resistance mean to you?”, “where do you get information about AMR from?,” “do you think antibiotic use in farming has any role to play in driving resistance in animals/humans?,” and “who do you think might have a role to play in addressing AMR?” For the full schedule, see Supplementary Material.

### Procedure

Prior to the interview, potential participants were sent a link to an online survey (hosted in Qualtrics) to be completed before their interview. The link contained the information sheet, consent form, and demographics questionnaire. Demographic data were collected for age, gender, and ethnicity. Farmers were asked to detail their highest level of education, number of years in farming, the nature of their farming system, and which livestock species they kept. Vets were asked to provide the year they qualified, details of any postgraduate qualifications, and the species they commonly worked with.

Participants were advised at the start of the interviews that SG was a trainee health psychologist and PhD student, and did not have a veterinary or farming background; this was so they knew they were not speaking to a vet or farmer. The aim was to encourage participants to speak freely about their antimicrobial use to someone who was external to veterinary and farming communities. Participants were also aware the study would contribute to SG's doctoral research, exploring antimicrobial use in UK agriculture (although participants were not advised of this study's focus until the debrief). The interview schedule guided the discussion, but questions were adapted throughout each interview, based on each participant's responses. After the interview, participants were debriefed and asked if they wanted to enter the prize draw.

### Data Analysis

Interviews were transcribed using an orthographic approach; additional field notes were not taken during interviews, and transcripts and the analysis were not provided to participants for further comment. Data were analysed inductively using the six-phase process of thematic analysis outlined by Braun and Clarke (46).

Phases one to three of this process involved transcription and repeated listening, generating and refining codes, and outlining initial themes. Coding and theme generation was performed by SG using NVivo for Mac (version 11) (QSR International Pty Ltd). The coherence and conceptual distinctness of these themes was reviewed and refined in phases four and five. The final stage, phase six, involved the report writing itself. The analysis involved a constant process of code and theme refinement, moving between phases two to six; SG led the analysis, with coding and themes continuously developed and refined through discussion with JO and HH.

The process of thematic analysis was conducted three times to generate three separate analyses. First, the vet interviews and farmer interviews were treated as independent datasets and analysed without reference to the other, to understand key issues within each profession. Next, the datasets were combined and analysed together to generate a final set of themes exploring similarities and differences between the two professions.

### Analytic Approach

A critical realist stance toward data collection and analysis was adopted. Critical realism is an epistemological position situated between realism and relativism; it reflects the tension that exists between attempting to access and study an objective reality, whilst acknowledging that data collected does not provide direct access to such a reality (47). A critical realist approach asserts that knowledge can be “discovered” through scientific methods, but the process of discovery and accumulation of knowledge is inevitably socially constructed (48). As thematic analysis is not rooted in any theoretical, epistemological, or ontological position (46), it can be suitably applied within a critical realist approach.

## Results

Participants were 13 private practice farm vets (six women, seven men) and 12 commercial livestock farmers (all men) from across England, Wales, and Scotland; all described their ethnicity as white. The age range of 10 vets was 24 to 58 years (median (*Mdn*) = 30.5); three vets declined to disclose their age. Farmers' ages ranged from 28 to 64 years (*Mdn* = 36.5). Vets had been qualified for between 1 and 34 years (*Mdn* = 7.5); three had postgraduate veterinary qualifications. Most vets (*n* = 10) described their current role as at the assistant or employee level, whilst three worked at partner or management level. All farmers described themselves as key decision-makers for antimicrobial treatments and were either employed as livestock unit managers or were running their own farms. They had been farming for between 4 and 49 years (*Mdn* = 16.0). Most vets worked predominantly with cattle and sheep, but some also worked with goats, poultry, horses, and small animals; one participant was a specialist pig vet. This reflects that most UK farm vets work predominantly with ruminants. Most farmers kept beef cattle, dairy cattle, sheep, or a combination of these; three farmers also kept either pigs or poultry. For individual demographic descriptions, please see Tables 1, 2. Pseudonyms are used for all participants.

**Table 1:** Demographic characteristics of veterinarian participants.

**Participant pseudonym**	Age (range)^*^	**Gender**	**Ethnicity**	**Years qualified (range)**	**Role level**	**Post-graduate qualification**	Species often working with^**^	Species sometimes working with^**^	Species rarely/never working with^**^
Richard	51–60	Male	White	31–40	Partner/Management	No	Pigs	N/A	Beef, dairy, goats, horses, poultry, sheep, small animals
Matt	21–30	Male	White	1–5	Assistant/Employee	No	Dairy	Beef, sheep	Goats, horses, pigs, poultry, small animals
Lisa	–	Female	White	1–5	Assistant/Employee	No	Beef, dairy, sheep	Goats, pigs	Horses, poultry, small animals
Hannah	21–30	Female	White	1–5	Assistant/Employee	No	Beef, dairy	Sheep	Goats, horses, pigs, poultry, small animals
Cathryn	31–40	Female	White	11–20	Assistant/Employee	No	Beef, sheep, small animals	Goats, horses, pigs, poultry	Dairy
David	21–30	Male	White	1–5	Assistant/Employee	No	Beef, dairy,	Sheep	Goats, horses, pigs, poultry, small animals
Andy	31–40	Male	White	6–10	Assistant/Employee	Yes	Dairy	Beef, sheep	Goats, horses, pigs, poultry, small animals
Philip	–	Male	White	11–20	Assistant/Employee	Yes	Dairy, sheep	Beef,	Goats, horses, pigs, poultry, small animals
Jenny	21–30	Female	White	6–10	Partner/Management	No	Beef, dairy, sheep	N/A	Goats, horses, pigs, poultry, small animals
Susie	–	Female	White	1–5	Assistant/Employee	No	Dairy, goats, sheep	Beef,	Horses, pigs, poultry, small animals
James	31–40	Male	White	11–20	Assistant/Employee	No	Dairy	Beef, sheep	Goats, horses, pigs, poultry, small animals
Gemma	21–30	Female	White	6–10	Assistant/Employee	No	Beef, dairy,	Sheep	Goats, horses, pigs, poultry, small animals
George	51–60	Male	White	31–40	Partner/Management	Yes	Beef, dairy, goats, sheep	Pigs	Horses, poultry, small animals

* Where no age is provided, participants declined to provide this information.

** *Species listed alphabetically. N/A = not applicable*.

**Table 2:** Demographic characteristics of farmer participants.

**Participant pseudonym**	**Age (range)**	**Gender**	**Ethnicity**	**Years farming (range)**	**Highest level of education**	Farming system^*^	**Farming purpose**	Species kept for commercial purposes^**^
Chris	31–40	Male	White	11–20	A-Level/Equivalent	Extensive	Food production; breeding; conservation or land management	Beef, sheep
Joe	31–40	Male	White	11–20	Undergraduate degree	Semi-intensive	Food production	Sheep
Mark	31–40	Male	White	6–10	Undergraduate degree	Intensive	Food production; breeding	Dairy cattle, sheep
Tim	61–70	Male	White	41–50	Undergraduate degree	Semi-intensive	Food production	Beef, pigs, sheep
Johnny	21–30	Male	White	6–10	Postgraduate degree	Semi-intensive	Food production	Beef, dairy cattle
Luke	31–40	Male	White	1–5	Undergraduate degree	Intensive	Food production	Beef, sheep
Nick	31–40	Male	White	1–5	Undergraduate degree	Intensive	Food production	Dairy cattle
Pete	31–40	Male	White	6–10	Postgraduate degree	Extensive	Food production; breeding	Sheep
Michael	41–50	Male	White	21–30	Undergraduate degree	Intensive / Semi-intensive / Extensive	Food production; education and research	Beef, dairy, pigs, sheep
Simon	31–40	Male	White	11–20	GCSE/NVQ/Equivalent	Semi-intensive	Food production; conservation or land management	Beef, sheep
Gavin	41–50	Male	White	31–40	No formal qualifications	Semi-intensive	Food production; breeding	Dairy cattle
Bill	61–70	Male	White	41–50	GCSE/NVQ/Equivalent	Semi-intensive	Food production; breeding	Beef, poultry (egg-laying)

* Self-categorized from pre-defined list; no farmers reported running an organic system.

** *Species listed alphabetically*.

**Figure 1:**
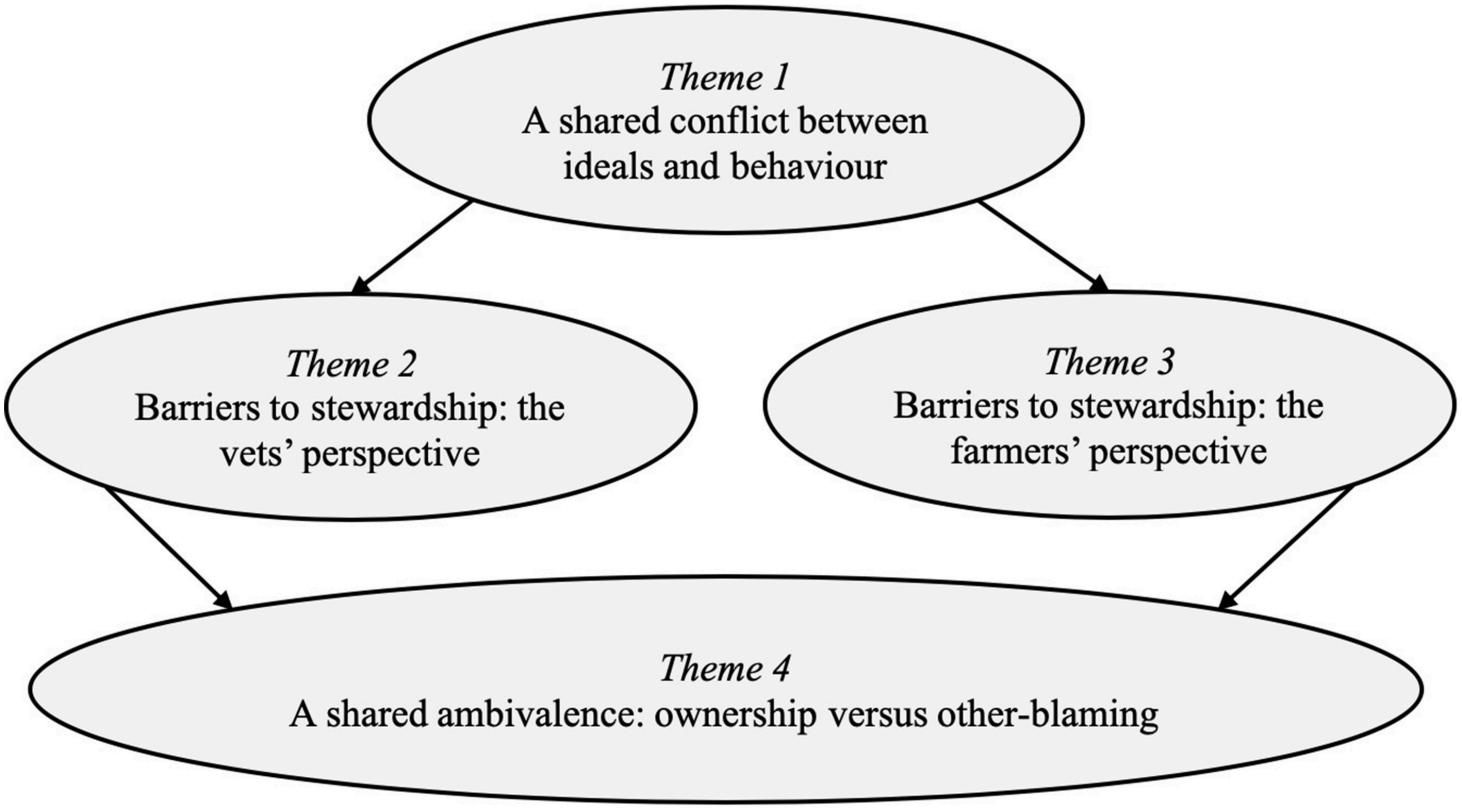
Overview of key themes.

The analysis generated four key themes, each with sub-themes, that explore the similarities and differences between vets' and farmers' beliefs about AMR and antimicrobial stewardship (for an overview of themes, see Figure 1). The first theme, “A shared conflict between ideals and behaviour,” highlights that vets and farmers have a shared understanding of the challenges posed by AMR and the need for increased antimicrobial stewardship, but do not always prioritize stewardship in their everyday treatment decisions. The next two themes, “Barriers to stewardship: the vets' perspective” and “Barriers to stewardship: the farmers' perspective,” focus on the key challenges that vets and farmers see as preventing them from prioritizing AMR and making further improvements to antimicrobial use on farms. The final theme, “A shared ambivalence: ownership vs. other-blaming,” highlights the ambivalent relationship that vets and farmers have with antimicrobial stewardship and responsibility for AMR. These themes will now be discussed using exemplar quotes.

### Theme 1. A Shared Conflict Between Ideals and Behaviour: “I Don't Like Using Antibiotics, but You Have to”

The first theme highlights vets' and farmers' beliefs about AMR and about their responsibilities for appropriate antimicrobial use, as well as their concerns about threats to animal welfare. It also highlights a conflict between vets' and farmers' knowledge about stewardship ideals and their own antimicrobial usage behaviour. There are three sub-themes: “Shared knowledge of stewardship,” “Shared anxieties for animal health,” and “Shared future discounting.”

#### Sub-theme 1.1. Shared Knowledge of Stewardship: “We've Gotta Be Responsible”

Both vets and farmers demonstrated a good awareness of the potential threats from AMR for human and animal health, and understood the risks posed by drug-resistant infections. In relation to farm animals, these risks were often framed in terms of threats to animal welfare, income, and productivity:

“*More animals would die, I think, as simple as that…they would not thrive as well”* (Simon, beef and sheep farmer).

“*If we did get an outbreak of mastitis that…you couldn't…contain, it could be a massive economic loss…I could lose thousands”* (Johnny, cattle farmer).

Antimicrobial stewardship was acknowledged as being a key part of the strategy to protect humans and animals from drug-resistant infections; vets and farmers both recognized the role their own antimicrobial usage played in this:

“*It's always in the back of your mind, every time you prescribe an antibiotic…am I selecting for resistance in any way?”* (Lisa, cattle and sheep vet).

“*Obviously we've gotta be responsible for what we're using, in the same way that human medicine's gotta be responsible for what they're using”* (Michael, cattle, sheep, and pig farmer).

At a conceptual level then, it was clear that vets and farmers both understood the potential risks from AMR and recognized their responsibility to minimize potential harms from inappropriate use.

#### Sub-theme 1.2. Shared Anxieties for Animal Health: “You're Gonna End up With Welfare Issues”

Despite this apparently high awareness of the risks from AMR and the need to be antimicrobial stewards, vets and farmers generally appeared not to perceive AMR as a current threat for their own practice and farms. With the possible exception of anthelmintic resistance, AMR was not something vets and farmers felt they had encountered for themselves:

“*You hear about it, and you read about it in the farming press…but I can't physically say I've seen it…nothing's ever been proven to me as we've had resistance to an antibiotic”* (Bill, beef farmer).

Instead of AMR being experienced by vets and farmers as a current threat, mostly they were concerned about their future ability to protect animal welfare and continue treating sick animals effectively. This was partly due to the risk of antimicrobials becoming ineffective if microorganisms developed resistance but was also due to concerns that antimicrobial use will become restricted in livestock. Vets and farmers stressed they did not want animals to suffer as a result of restricted treatment options, but opinions were mixed regarding tighter regulation:

“*We need to acknowledge there are antibiotics that for whatever reason have become of critical importance to the human field, and their use should be, in my opinion, limited”* (Richard, pig vet).

“*It would be good for antibiotic choice…however…it would be another nail in the coffin for the farming industry”* (Hannah, cattle vet).

Linked to these concerns that their ability to protect animal welfare might be undermined in the future, vets and farmers felt a shared frustration that antimicrobial use in livestock is being unfairly targeted as a key driver of AMR, and that greater stewardship efforts were needed in other domains. There was concern that the public narratives about antimicrobial usage in farming could have negative impacts on animal welfare:

“*The press like to make it look like we're causing the problems…it's good PR for the government, or whoever's in power, to blame the farmers for using antibiotics”* (Chris, beef and sheep farmer).

“*You're gonna end up with welfare issues [if] you're not treating a sick animal…that's where sadly this whole argument gets a bit hijacked by government and by people that are anti-antibiotic use…I'm sorry, but there is a need for antibiotics”* (Michael, cattle, sheep, and pig farmer).

Vets and farmers therefore understand the theoretical future risks from AMR, including threats to human and animal health, but in terms of their everyday experiences, their immediate concerns relate to potential regulatory threats to treatment options. They are worried that antimicrobial use in farm animals may be restricted, resulting in situations where sick animals cannot receive required treatments, leading to poorer animal welfare.

#### Sub-theme 1.3. Shared Future Discounting: “I'm Sure We all Have This Wonderful Ideal”

Vets and farmers often spoke at length about potential risks from AMR, but those risks appeared not to be salient enough in the here-and-now of their everyday decision-making. AMR mostly represents a future threat to vets and farmers, a threat they often discount in the face of more immediate concerns. Therefore, despite vets and farmers having good awareness of potential risks from AMR, a factoring of these risks into their treatment decisions was not always evident. Immediate concerns, such as welfare and productivity, were recognized as more pressing factors:

“*It's not something you get up in the morning and think, oh, antibiotic resistance today…most farmers' agendas are trying to financially keep the place on an even keel and keep their stock as productive and healthy as possible”* (Tim, beef, sheep and pig farmer).

There was also evidence that the salience of the risks of driving AMR from inappropriate use of antimicrobials varied between different decisions. For example, although most vets reported “making a conscious effort to…avoid certain classes of antibiotics” (Gemma, cattle vet), others acknowledged that AMR concerns cannot always be prioritized and are “just something else to be weighed up against all the other factors” (David, cattle vet). Vets appear to adjust their prescribing, sometimes at the expense of stewardship principles, to account for the specific context:

“*I'm sure we all have this wonderful ideal of what we would like to do…[but] putting it into practice can sometimes be a bit trickier”* (Gemma, cattle vet).

Farmers also alluded to a tension between wanting to avoid antimicrobial usage and finding themselves in situations where they felt there was no alternative:

“*I'm more prevention, than cure…but…I have to safeguard and protect my animals for profitability and fitness and also welfare…I don't like using [antibiotics], but you have to”* (Gavin, dairy farmer).

This theme therefore begins to illustrate the conflict that vets and farmers experience between knowing they should be antimicrobial stewards and knowing that their own prescribing and usage behaviour is not always aligned to stewardship ideals. To explain this conflict between their ideals and behaviour, vets and farmers discussed the importance of contextual factors on their antimicrobial usage decisions. Despite some commonalities between the professions, different factors appeared to represent greater challenges to vets or to farmers. Exactly how these contextual factors influenced their decisions and acted as barriers to antimicrobial stewardship will be discussed across the next two themes.

### Theme 2. Barriers to Stewardship: The Vets' Perspective: “You Have to Be Fairly Confident in Your Client Relationship”

The second theme describes how vets' treatment decisions are influenced by their perceptions of the situational factors on-farm, differences between the farmers themselves, and concern about maintaining client satisfaction and protecting their relationships with farmers. There are three sub-themes: “Situational factors,” “Farmer variability,” and “Relationship management.”

#### Sub-theme 2.1. Situational Factors: “Treatments…They're Farm Specific”

Disease was acknowledged to be influenced by farm-specific factors, including infrastructure quality and animals' underlying health status. Vets felt the most appropriate treatment varied across farms, farming systems, and industries, and they stressed the importance of understanding the farm's disease and treatment history when assessing treatment options:

“*Treatments…might be generic [but]…they're farm specific really. You need to know what's going on, on the farm, to be able to advise them”* (Philip, dairy and sheep vet).

Uncertainty in determining the causal agent was regularly mentioned as driving antimicrobial use in situations where antimicrobials were not definitely needed. Sometimes, uncertainty was managed by prescribing antimicrobials:

“*There's always the fear…[with]* E. coli *mastitis, there's evidence to suggest you don't really need to prescribe an antibiotic, but quite often we do, and…I don't think I'd be brave enough not to prescribe an antibiotic”* (Lisa, cattle and sheep vet).

Vets wanted to make greater use of diagnostic testing to reduce inappropriate prescribing, but felt this was often impractical, with time delays the most commonly cited barrier:

“*If you've got an acutely ill animal…you can't really sit and wait for 48 to 72 hours to grow something on a plate”* (David, cattle vet).

Vets were also sensitive to financial pressures faced by farmers, which they felt limited their ability to help farmers reduce inappropriate antimicrobial use. For example, concerns were raised about short milk withdrawal periods on third and fourth generation cephalosporins, which presented vets with an economic challenge to prescribing alternative drugs:

“*It's harder to say…I think we should go for a penicillin because it's a more responsible choice, but that means you're not gonna be able to put milk in the tank”* (Lisa, cattle and sheep vet).

Nonetheless, vets also reported sometimes using economic arguments to persuade farmers to agree to alternative treatments, for example by presenting economic benefits of disease prevention:

“*By managing, preventing, reducing lameness in sheep flocks, there's a reduction in antibiotic usage [and]…huge welfare benefits and performance benefits”* (Susie, dairy, sheep, and goat vet).

Vets' judgements about the most appropriate treatment can therefore shift according to their perceptions of the specific situation on-farm.

#### Sub-theme 2.2. Farmer Variability: “Just Because of the Type of Farmer”

Vets' prescribing is also influenced by the varying knowledge, abilities, and personalities of farmers. Mostly, vets felt farmers followed their treatment advice, but if they were concerned a farmer might not adhere to treatment plans, they would account for this when prescribing:

“*A particularly diligent stockman…we might just ask them to inject. If we felt they were gonna miss half the cases, then we might prefer to blanket medicate”* (Richard, pig vet).

Vets were aware that farmers are busy people and they sometimes prescribed long-acting medications to reduce the farmer's workload. Long-acting medications were also sometimes prescribed for other practical reasons, such as ease of administration, or farmer health and safety:

“*I didn't want him to get killed trying to comply [laughs]…[the cow] was nuts, she was really angry”* (Jenny, cattle and sheep vet).

Perceived differences in farmers' personalities was acknowledged as sometimes influencing vets' prescribing decisions:

“*Alamycin, I'd probably suggest giving two courses. For an impatient farmer, I suggest giving one course and then moving onto another drug just because of the type of farmer that he is”* (Hannah, cattle vet).

Farmer personality also influenced vets' willingness to raise the topic of antimicrobial stewardship and discuss alternative drugs or preventive measures, and there was frustration at those farmers who appeared resistant to change:

“*The trouble is…farmers that are doing it wrong will not attend any courses or listen to anything you've got to say”* (Cathryn, beef and sheep vet).

Vets' prescribing ideals are therefore challenged, and their treatment decisions influenced by, the individual differences they perceive between farmers.

#### Sub-theme 2.3. Relationship Management: “You Can End up Having a Little Row”

Vets felt farmers had a lot of respect for them, but it took time for them to develop an effective relationship with farmers:

“*You need to build up your repertoire with the farmers before they'll pay any attention to you”* (Matt, dairy vet).

Central to some prescribing choices by vets was an underlying need to manage their own, and farmers', emotions, particularly in situations of clinical uncertainty. Vets were concerned about negative outcomes for the farmer, the animal, and their own reputations. They were especially concerned about actively refusing treatment or recommending alternative antimicrobials:

“*Sending someone away with no treatment is not easy, you have to be fairly confident in your client relationship…if they can't accept it, they'll either go down the road for a second opinion or come back and say I don't want to see that useless vet who didn't give my animal any treatment”* (George, cattle, sheep, and goat vet).

Although vets did discuss ambiguous clinical situations when they chose not to prescribe, sometimes the potential risk, and associated emotional cost, was just too great. Occasionally, vets felt farmers just expected them to do something and they acknowledged the psychological benefits from the act of treating, even when treatment might be inappropriate:

“*Even though if you didn't give the antibiotic [the animal] would have looked better, it gives [farmers]…peace of mind that they've done something to try and help that animal”* (Hannah, cattle vet).

Vets acknowledged their professional responsibility to prescribe appropriately and knew they should challenge farmers' expectations surrounding antimicrobial treatment. Nonetheless, vets admitted they sometimes prescribed antimicrobials to avoid or resolve awkward consultations:

“*Someone who's like, well I think it needs this, and you're like, well I think it doesn't…you can end up having a little row and…for the sake of an easy life, sometimes you're just like fine, fine, you're wrong, but fine”* (Gemma, cattle vet).

Furthermore, there was a sense that there needed to be greater consistency of stewardship messages communicated to farmers by the veterinary profession. Some vets admitted they would sometimes prescribe against their own judgement because they suspected their vet colleagues would override their decision, undermining their relationship with their farmer clients:

“*Knowing that actually, if I say no, that farmer would just phone one of the other vets, and they'd say yes. So that's quite hard, to make a responsible decision”* (Lisa, cattle and sheep vet).

This theme therefore highlights some of the barriers that vets perceive as preventing everyday antimicrobial stewardship. Vets' treatment decisions are influenced by farm-specific factors, such as herd health status or economic concerns. Vets also adjust their treatment decisions to account for farmers' abilities or personalities. Finally, vets are concerned about maintaining their relationships with their farmer clients and keeping them satisfied. Although vets are concerned about AMR, the here-and-now challenges of their treatment decisions often take priority over their intentions to act in line with stewardship ideals.

### Theme 3. Barriers to Stewardship: The Farmers' Perspective: “Putting Pressure on the Cost of Production”

The third theme describes how farmers' treatment decisions are made within a context that is constrained by financial concerns and industry pressures. Farmers' decisions are also influenced by the messages, sometimes conflicting, that they receive about antimicrobial usage from vets and the government. There are three sub-themes: “Economic challenges,” “Competing industry drivers,” and “Conflicting messages.”

#### Sub-theme 3.1. Economic Challenges: “The Money's Not There in the Job”

Throughout their discussions of antimicrobial usage, farmers provided examples where financial factors either promoted or prevented antimicrobial usage in line with stewardship ideals. Although farmers understand the need to use antimicrobials responsibly, they also face real practical issues, such as limited time and money, which they feel prevent them from making further changes to improve health status and prevent additional usage of antimicrobials:

“*The money's not there in the job at the minute…people are getting paid less than cost of production…everyone's trying to cut corners where they can to save money and there's gonna be repercussions because of it”* (Johnny, cattle farmer).

Although they generally trusted the veterinary advice they did seek out, some farmers felt vets didn't have much of a role in antimicrobial stewardship on-farm, because veterinary services were too expensive:

“*[Vets] cost money…as soon as you have a conversation of a management matter, it's a hundred pounds or something ridiculous like that”* (Simon, beef and sheep farmer).

There were also negative views expressed about potential legislative changes that might restrict a farmer's right to administer antimicrobials. There was a sense from farmers that this would both threaten their livelihoods, and challenge their ability to perform their job effectively:

“*[If] every sick pig's gotta be looked at by the vet…if you get to that stage…you might as well write-off British livestock agriculture”* (Michael, cattle, sheep, and pig farmer).

Measures to improve antimicrobial use on-farm were therefore sometimes seen as posing an additional economic burden on farmers, especially for those businesses already struggling to remain profitable.

#### Sub-theme 3.2. Competing Industry Drivers: “Food With Less Antibiotics Will Be More Expensive”

Linked to the cost-benefit consideration of each individual decision were broader industry and consumer pressures, which farmers felt could drive both good and bad practice. Farmers acknowledged the benefits of industry-led initiatives and recognized the potential power of consumers to drive improvements in antimicrobial usage; they were increasingly aware of stewardship messages coming from commercial product-buying organizations and farm assurance schemes:

“*The retailers don't…want us to be blanket treating them [the cattle] with antibiotics any more”* (Luke, beef and sheep farmer).

Despite welcoming some industry-led initiatives, farmers also felt frustrated and constrained by the economics of the industries and systems they worked in. There was a sense that industry and consumer demand for cheap meat and milk products was preventing further improvements on many farms. Some farmers felt the demand for cheap produce was linked to poorly managed systems that could increase the need for antimicrobials; sometimes, this demand for cheap produce was also linked to concerns about intensification:

“*With people looking for cheaper and cheaper food…that's putting pressure on the cost of production…so people do take shortcuts and keep animals in unsuitable accommodation and so on”* (Mark, dairy and sheep farmer).

“*If we want to go down this line of intensive farming, for cheap meat, then I don't see any other way of doing it…you're gonna get these big problems, because everything's done on a big scale, and therefore you're going to need the antibiotics”* (Joe, sheep farmer).

Supermarkets and other produce buyers were therefore seen as having a pivotal role in driving current farming practices and associated antimicrobial usage. There was a desire for farmers to receive better financial support in order to continue driving change, which most felt should come in the form of a better price for farmers' produce:

“*Supermarkets seem very reluctant to tell the consumer how it is…the consumer needs to face, be given the choice of cheap food or sustainable food, and obviously food with less antibiotics in it will be more expensive”* (Nick, dairy farmer).

Farmers were therefore aware of the stewardship messages coming from industry stakeholders. Nonetheless, they felt constrained by what they perceived as prevailing market forces, that keep the purchase price of products too low for many farmers to re-invest and improve their management systems.

#### Sub-theme 3.3. Conflicting Messages: “I Don't Think the Vets Are all on One Hymn Sheet”

Farmers generally reported getting their information and support about AMR and stewardship from vets, industry bodies, and government, and were sceptical of the messages about AMR they heard in the mass media. Vets were seen as a valuable and credible source of information regarding AMR and antimicrobial stewardship, and farmers appeared to welcome vets' input on these issues:

“*[Vets] are the go-to people for advice, and they are…well-educated experts…if they said something to a farmer, I think it would make a farmer stop and listen”* (Nick, dairy farmer).

Farmers also felt there was a role for governmental and industry bodies to lead on improving antimicrobial usage on farms, and such bodies were regularly mentioned as having a central role in disseminating stewardship messages. There was a belief amongst farmers that these bodies had the resources and expertise to conduct the required research and provide evidence-based guidance to each specific livestock industry:

“*I tend to take everything in the press with a pinch of salt…[if] the VMD*^1^ *or APHA*^2^ …*National Pig Association…or if AHDB*^3^ *come out with something then I tend to take more notice of that, because it should be based on science and fact and evidence”* (Michael, cattle, sheep, and pig farmer).

Despite this general faith in veterinary and governmental advice, there was, however, some evidence that farmers felt they received conflicting messages from these sources. For example, farmers perceived variation in the advice they received from vets regarding antimicrobial treatment options:

“*We've had two conflicting…veterinary opinions…I don't think the vets are all on one hymn sheet, are they?”* (Joe, sheep farmer).

Furthermore, there was also some scepticism amongst farmers about the government's ability to co-ordinate effective animal health campaigns:

“*You could say DEFRA*^4^*, but when you see the shambles they're doing on the TB [tuberculosis] malarkey, whether or not they'd be the right ones to do that, I don't know”* (Bill, beef farmer).

Receiving conflicting messages from the sources they perceive to be otherwise credible could undermine the trust that farmers place in vets and the government. If key stewardship messages are perceived as inconsistent, farmers may also be less motivated to change their antimicrobial usage.

This theme therefore highlights some of the barriers farmers felt prevent them from making further improvements in their antimicrobial usage. Farmers felt constrained by external forces, such as economic challenges and industry pressure. Furthermore, they reported getting conflicting signals from key messengers, potentially undermining those messengers' efforts to encourage farmers to change their behaviour.

### Theme 4. A Shared Ambivalence: Ownership vs. Other-Blaming: “I Want to Reduce…the Risk of Antibiotic Lottery”

The first three themes therefore illustrate the conflict vets and farmers experience between their ideals about stewardship and their own, less-than-ideal behaviour in some situations. It is clear vets and farmers understand their responsibilities as antimicrobial stewards, but they perceive various barriers that prevent them from always using antimicrobials in line with stewardship principles. The final theme explores how these tensions manifest for vets and famers as a sense of ambivalence toward their antimicrobial stewardship responsibilities: vets and farmers express both ownership for stewardship and other-blaming for inappropriate antimicrobial use. There are two sub-themes: “Shared ownership” and “Shared other-blaming.”

#### Sub-theme 4.1. Shared Ownership: “You Think of the Sort of Moral Issues”

It is evident that vets and farmers know they should practice good antimicrobial stewardship. Disease prevention was seen as a key strategy for reducing antimicrobial usage, and all vets and farmers agreed with the benefits of preventive measures, such as vaccinations, improving housing, and genetic breeding to strengthen natural immunity. Avoiding sick animals in the first place was a primary aspiration for all farmers:

“*If you're giving the antibiotic…you're losing growth or whatever because the animal isn't thriving, so we try and keep the animals healthy rather than have to treat them”* (Chris, beef and sheep farmer).

Vets expressed a clear sense of ownership in promoting stewardship to farmers, and felt it was especially important that they worked with farmers to improve farmers' treatment decisions:

“*I want to reduce…the risk of antibiotic lottery, where farmers don't know what they should be using…[now] they've got a standard operating procedure to work from”* (Susie, dairy, sheep, and goat vet).

Both vets and farmers recognized the complexity of decision-making surrounding antimicrobial treatment, but farmers more often saw their diagnostic decisions as straightforward. Compared to vets' decisions, farmers' diagnostic decision-making appeared rather binary; an animal was either sick or it wasn't, and sick animals needed antimicrobials. Vets felt farmer education in this area was a key part of their own role as antimicrobial stewards and they discussed ways they worked with farmers to better manage ambiguous cases:

“*We teach them pattern recognition, to know what's appropriate to treat, and what's not”* (Richard, pig vet).

Farmers also expressed a sense of ownership for improving antimicrobial usage and recognized that in some cases using antibiotics would not necessarily confer any benefits to the animal. They discussed various examples of when they withheld antibiotics, such as when they suspected the cause might be viral, when they felt pain relief was sufficient, or when they simply felt through experience that antibiotics would make little difference to the outcome:

“*If the symptoms are already on the wane…a shot of antibiotics is probably not going to do too much, other than make you feel better, rather than the sheep [laughs]”* (Pete, sheep farmer).

Furthermore, although farmers reported administering blanket treatment, they were aware it was not always necessary and could drive the development of drug-resistant pathogens. Some farmers discussed how the threat of AMR was motivating them to make changes to try and reduce this type of treatment:

“*Alamycin…I used that as a blanket treatment [for enzootic abortion] and it stopped it. Great. But then you think of the sort of moral issues with it, so I've started to…put the vaccine into my young ewes”* (Joe, sheep farmer).

Taking ownership for antimicrobial stewardship was seen by both vets and farmers as being a joint enterprise between both professions; encouraging collaborative working between vets and farmers was considered an important strategy for improving stewardship. There was a sense that shared knowledges and experiences from the wider veterinary and farming communities informed treatment decisions; finding ways to share success stories was one strategy that vets used to support farmers in making changes to antimicrobial use:

“*Farmers that have done it and had success…it's about getting them together…to share their experiences”* (James, dairy vet).

Breaking habits was recognized as hard, but vets did discuss examples of when they had managed to facilitate changes on-farm through discussion with farmers. There was also a suggestion that vets' own perceptions of their farmer clients could be wrong. If they could find a way to engage a particular farmer and understand their needs, vets could have an unexpected impact on farmer behaviour and successfully reduce antimicrobial use:

“*One farm…would've been inappropriate use of fluoroquinolones…we managed them onto a penicillin-based product…[and] a respiratory vaccine…He's not someone you'd expect would be so easy, but he's been a very easy example”* (Andy, dairy vet).

Both vets and farmers could be wary of change; they acknowledged that behaviour change can be difficult, but recognized change was needed to improve antimicrobial usage. Farmers mostly felt vets were the experts who could provide them with support and motivation to reduce antimicrobial use on-farm, and farmers wanted their vets to guide them:

“*I was very, very, very sceptical about this selective dry cow therapy…because my vet told me not to do it…he's had experience of people doing it and it didn't work, but then…me and the vet, we went to a…seminar [about]…mastitis research…and he changed his tune overnight, right there and then…so then I've given it a go”* (Johnny, cattle farmer).

It is clear then that vets and farmers understand their responsibilities as antimicrobial stewards and understand the need to change their practices to improve antimicrobial usage in the animals they work with.

#### Sub-theme 4.2. Shared Other-Blaming: “The Steps That I go to”

Despite this clear sense of ownership for antimicrobial stewardship, however, there was also some scepticism expressed by vets and farmers about the threat posed by agricultural antimicrobial use, especially in relation to human health:

“*We would argue the vast majority of antimicrobial resistance is actually created within the human population, and the animal contribution…is actually relatively small”* (Richard, pig vet).

This scepticism means that, alongside a sense of ownership for the problem, vets and farmers also engaged in other-blaming for rising rates of AMR. This other-blaming happened at different levels, both within and without the UK, and was directed at various groups across both veterinary and human medicine. Both vets and farmers feel frustrated that their own stewardship efforts are being undermined by the actions of other key stakeholders, and there were frustrations at differing practices across the global community:

“*Other countries are using antibiotics willy-nilly…we can do as much as we can over here, but then is it gonna make much of a difference, unless the other countries do something as well?”* (James, dairy vet).

Much of this other-blaming by vets and farmers was directed at human medicine. There was a common feeling that stewardship in human medicine was insufficient, and vets and farmers regularly discussed inappropriate use by doctors and patients:

“*The steps that I go to, to make sure my sheep flocks complete a course of antibiotics, but actually how many people…don't complete courses”* (Susie, dairy, sheep, and goat vet).

As well as directing their frustrations at human doctors and patients, vets and farmers also directed some of this other-blaming toward the current state of the UK farming industry. For example, vets and farmers acknowledged that although in principle more could be done to prevent disease, many changes were beyond farmers' financial capabilities. Some also felt that levels of antimicrobial usage were intrinsically linked to the nature of some farming systems:

“*I don't think you can manage without antibiotics in the kind of system that I'm doing here, certainly with the cattle anyway, which is a shame, but I can't see how you could possibly do it without it”* (Luke, beef and sheep farmer).

Finally, as well as directing blame at human medicine or the farming industry, vets and farmers also felt there was room for improvement amongst their own veterinary and farming colleagues. Sometimes, this other-blaming was directed at vets:

“*I'm very careful about my use of fluoroquinolones…which some vets seem to dish out willy-nilly”* (George, cattle, sheep, and goat vet).

“*It's down to them at the end of the day, isn't it…I mean, I'm only a farmer aren't I…I'm relying on the vets…to tell us which are the best ways to go”* (Gavin, dairy farmer).

At other times, farmers were the target for this other-blaming:

“*I know for a fact there are farmers out there…doing naughty things, which means antibiotic milk is getting into the system”* (Nick, dairy farmer).

“*We're doing it at the right dose, the right route…so many farmers…are giving an inappropriate drug, an inappropriate dose”* (Cathryn, beef and sheep vet).

Therefore, despite the desire for collaborative working evidenced in the previous sub-theme, there was also evidence that sometimes vets and farmers felt frustrated at what they felt was less good behaviour by colleagues.

This final theme therefore illustrates that vets and farmers appear to have an ambivalent relationship with antimicrobial stewardship and responsibility for AMR. Whilst they understand they need to take ownership for stewardship, vets and farmers also engage in other-blaming for the problem of inappropriate antimicrobial usage. Vets and farmers therefore appear to feel that their stewardship efforts are undermined by the actions of other key stakeholders, including those of other vets and farmers.

## Discussion

This in-depth qualitative study identified that farm vets and farmers who participated share a good awareness of potential risks from AMR and of their roles as antimicrobial stewards. It is clear, however, that various psychological and contextual factors influenced participants' beliefs about their stewardship responsibilities, and that these factors prevent these vets and farmers from always acting in line with their own stewardship ideals when making antimicrobial treatment decisions. The analysis highlighted a difference between the barriers that loomed largest for either vets or farmers. Vets are especially influenced by situational factors on-farm, variability in farmers' personalities, and a need to manage their relationships with clients and colleagues. Farmers feel constrained by economic challenges, competing industry drivers, and conflicting messages from the government and from different vets.

The interplay between psychological and contextual factors is highlighted by the importance of emotional and social influences on vets' and farmers' treatment decisions. Sometimes vets acted upon client pressure to prescribe, even when they felt antimicrobials were not necessary; similar pressures have been reported elsewhere in both veterinary and human medicine (4, 13, 49). A potential lack of support from veterinary colleagues for prescribing decisions was an issue for some vets in this study, who sometimes prescribed less responsible antimicrobials to avoid being undermined by colleagues. Comparable concerns about a lack of professional support for prescribing decisions have been identified amongst cattle vets (29) and junior hospital doctors (50). This lack of consistency with stewardship principles between vets may be partly responsible for the conflicting veterinary messages reported by farmers in this study. Finally, prescribing to manage clinical uncertainty and fear of negative outcomes sometimes contributed to both vets' and farmers' inappropriate antimicrobial usage. They are not alone in this; doctors also report that in situations of uncertainty they are more inclined to over-treat with antimicrobials, rather than withhold treatment (4, 51).

Vets and farmers in this study also expressed scepticism about the agricultural contribution to AMR, especially regarding potential links between agricultural antimicrobial use and risks to human health, adding to similar findings from other veterinary contexts (28, 31, 32, 52). Despite controversy surrounding the exact pathways of resistance between animal and human bacterial populations, there nonetheless remains the risk of animal-to-human transfer of resistance genes (1), and vet or farmer scepticism of this risk is a potential barrier to stewardship. Indeed, this scepticism may partly contribute to the gap between the stewardship ideals that vets and farmers discussed and their ‘less-than-ideal' everyday treatment decisions.

This shared gap between vets' and farmers' knowledge and behaviour suggests they hold competing beliefs about what constitutes appropriate usage in a given situation; this psychological gap can be considered a form of cognitive dissonance (53). As a result of these tensions, vets and farmers in this study appear to have an ambivalent relationship with the concept of antimicrobial stewardship. Whilst they recognize their own roles as antimicrobial stewards, vets and farmers also feel frustrated at what they perceive to be bad practice elsewhere. This frustration results in other-blaming, with both vets and farmers laying the blame for increasing AMR and bad practice on other parties, including other vets and farmers, doctors and patients, and other antimicrobial users across the globe. Vets and farmers are not, however, the only groups to other-blame and locate the issue of AMR elsewhere; surveys of doctors in Ghana and Jamaica have shown they consider AMR to be of greater threat to global or national communities as compared to their local communities or institutions (54, 55).

### Implications for Research, Policy, and Practice

Identifying ways to overcome this ambivalence and other-blaming, to encourage more collaborative working between vets and farmers, could be a potential focus for future stewardship interventions. If individuals lay the blame for inappropriate usage with others, they will be less likely to critically reflect on whether there is room for improvement in their own practice. Change will, however, be needed at different levels. Other-blaming is a psychological factor influencing these vets' and famers' treatment decisions, but this other-blaming is related to the contextual, day-to-day challenges that vets and farmers describe facing. It is a false dichotomy, however, to distinguish between the individual and their external world, as in practice individual and interpersonal (psychological) factors exist within a reciprocal dynamic with external (contextual) factors. A social ecological approach emphasizes the need to recognize both psychological and contextual factors, as well as the dynamic interplay between them (11, 12). From this perspective, interventions to increase stewardship will need to acknowledge this reciprocity between individuals and their contexts. Potential approaches that could inform interventions will now be considered at the individual-, group-, and societal-level, as successfully promoting antimicrobial stewardship in farming will likely require interventions at all three levels. Although psychological factors are generally targeted at the individual- or group-level, and contextual factors at the group- or societal-level, all interventions can have wider impacts upon both psychological and contextual factors. Changing an individual's or group's psychology can alter their context, and changing context (at either a group- or societal-level) can impact an individual's or group's psychology (10–12).

#### Individual-Level Change

An individual-level approach to overcoming other-blaming would be to increase the use of inclusive, One Health approaches to stewardship awareness campaigns that target individuals' knowledge and motivations. Vets and farmers can feel blamed and stigmatized by others for AMR (33, 56), so inclusive stewardship campaigns, such as “Antibiotic Guardian,” may be more acceptable to vets and farmers (57). These awareness-raising campaigns are limited, however, as they are unlikely to be very salient in everyday decision-making contexts, and therefore may have limited impact on behaviour change. Awareness campaigns are necessary but not sufficient for increasing stewardship, as they offer little in terms of practical solutions for prioritizing stewardship principles over more immediate concerns.

#### Group-Level Change

A group-level approach, based on social identity theory (58) and self-categorization theory (59), would be to enable vets and farmers to adopt a common ingroup identity (60) that is superordinate to their individual professions. Results from this study suggest vets and farmers are generally identifying within their own professions and blaming other professions for poor stewardship and increasing AMR. It would be counter-productive in the wider context of AMR to encourage vets and farmers to adopt a shared identity based on directing blame outside of farming (e.g., at medics, patients, the media), as this would likely decrease all groups' motivation to critique and improve their own behaviour, as well as increase division between groups that ultimately need to co-operate to tackle AMR. Instead, vets and farmers could be brought closer together by focusing on an emerging common fate; that access to antimicrobials may be restricted (either through legislation or because they become ineffective) unless action is taken to improve stewardship within livestock farming. Increasing the salience of a common fate can strengthen a shared social identity, which can then drive co-operation to achieve shared goals (59, 60). By bringing vets and farmers together to work on stewardship issues they may begin to adopt a shared group identity, perhaps termed “livestock professionals” (e.g., people who care about animal welfare and want to protect antimicrobials for that purpose). By increasing the salience of the new common ingroup identity, and reducing the salience of the specific profession identity, the motivation to lay blame for poor practice with members of the other group will likely diminish and the motivation to collaborate toward the common goal of improved stewardship will increase. Furthermore, by bringing together people with multiple perspectives, better animal health outcomes may be achieved; use of multi-disciplinary teams in human healthcare can improve patient outcomes (61, 62).

Improving stewardship on farms is inevitably going to require more collaborative working between vets and farmers, and future interventions could draw on a social identity approach (63) to increase communication and collaboration between these groups. A social identity approach can offer an alternative approach to developing health interventions by considering group-level processes, rather than focusing on individual-level processes (63). Indeed, emerging evidence suggests collaborative working between vets and farmers can be beneficial in developing antimicrobial stewardship policies for farms (19). Reductions in antimicrobial usage can also be achieved through collaborative working between vets and farmers, through changes to treatment protocols and tailored biosecurity and herd management interventions, without harming production parameters or animal welfare (16, 18). By co-creating solutions, the emphasis for locating responsibility for change with individuals may be reduced, and vets and farmers will be maximally invested in driving change together.

This collaborative approach may, however, need to be led, or at least initiated, by the veterinary profession. The insights from this study suggest that whilst farmers are strongly motivated to prevent disease through preventive medicine and better husbandry, they are likely to need further veterinary guidance on how to achieve best practice in antimicrobial usage. There appears to be scope for vets to be more proactive about driving preventive medicine on farms to improve welfare and reduce antimicrobial usage (64). They may need to adapt their communication styles and overcome their assumptions about some clients, to ensure engagement with all farmers about disease prevention and antimicrobial stewardship, including those farmers that vets perceive, perhaps incorrectly, as hard-to-reach (65).

UK-based vets may feel their efforts to promote antimicrobial stewardship have minimal impacts within a global context, but it could be argued that the UK veterinary profession can take a leadership role on this issue. The contextual barriers that vets perceive on farms represent a genuine challenge to antimicrobial stewardship, but these challenges (or at least some of them) should not be seen as insurmountable, given the right support. There is growing global momentum for antimicrobial stewardship, presenting an opportunity for the UK veterinary profession to take action and lead by example. Future research should therefore explore ways of fostering leadership and communication skills in vets, enabling them to help drive change, even within existing constraints.

#### Societal-Level Change

Improving antimicrobial usage in livestock will, however, require more than just interventions targeted at psychological factors located within and between vets and farmers; societal-level change, such as at the state- or industry-level, will also be required to alter the context within which vets and farmers make their treatment decisions. Indeed, it has been argued that locating responsibility for change solely with individuals minimizes the responsibility that lies with bodies such as governments or organizations (66, 67). The economic challenges and industry-related constraints discussed by vets and farmers in this study are real, and are important drivers of antimicrobial use across veterinary contexts (25, 27, 31). Farmers in this study related these challenges and constraints to the increasing demand for cheap produce; overcoming this demand will require a public debate about how meat and milk are produced and what people are willing to pay for produce. Potentially there is a need for public education about the challenges to sustainability associated with different approaches to farming. There are, however, ethical tensions between the need to produce sustainable meat, which should include the sustainable use of antimicrobials, and the issue of access to affordable sources of protein.

AMR, much like climate change, is often perceived as a future threat and a risk for other people, and appropriate antimicrobial usage can be considered a classic “tragedy of the commons” problem (9, 68). In the majority of cases, AMR is not salient enough in vets' and farmers' everyday treatment decisions; immediate risks and concerns arising from contextual pressures and constraints generally outweigh concerns about AMR. Society will need to find a way of constructively incentivizing vets and farmers, to make appropriate usage of antimicrobials more salient in the here-and-now, perhaps using financial incentives, such as those recently used in the English National Health Service (69). Debates will also be needed about what societal-level interventions might be both effective and acceptable to various stakeholders, including policy-makers, vets and farmers, and consumers. As with any intervention, societal-level changes could bring unintended consequences; levers such as legislation, professional obligations, and contractual requirements may reduce antimicrobial usage but could also drive poor husbandry if poorly implemented or only considered in isolation. Without also tackling economic constraints, social norms and narratives around food and farming, and the salience of immediate concerns over AMR in everyday treatment decisions, significant behaviour change in this area will remain challenging.

### Conclusions

The application of a psychosocial approach, using critical incident methodology, to explore vets' and farmers' antimicrobial treatment decisions identified that whilst they understand their responsibilities for antimicrobial stewardship, psychological and contextual factors, such as economics, emotions, and relationships appear to be important potential barriers to consistent stewardship-aligned decisions. The results suggest that, for vets and farmers in this study, a conflict between their ideals and behaviour leads to a sense of ambivalence toward their responsibilities for antimicrobial stewardship; they take ownership of the issue, but also engage in other-blaming and locate responsibility for AMR with others. The results also suggest that vets and farmers share and understand common challenges, some of which are also an issue for human medicine. AMR has been described as the “quintessential planetary One Health challenge” [35, p.508] and recognizing shared challenges (a common fate) between different groups may play a key role in improving antimicrobial stewardship.

Future research to develop stewardship interventions could utilize a social ecological approach, recognizing the interplay between psychological and contextual factors (11, 12), to consider ways to overcome the potential obstacles of other-blaming and ambivalence for responsibility for AMR. Societal-level change will be vital in tackling increasing rates of AMR, but even within the existing context there is room for interventions targeting professional groups. A social identity approach to reducing other-blaming could involve the development of a common ingroup identity between vets and farmers, emphasizing both their common fate as livestock professionals facing potential restrictions to antimicrobials, and their common goal of promoting animal welfare by optimizing antimicrobial stewardship on farms within existing economic and industry-related constraints.

## Ethics Statement

This study was carried out in accordance with the recommendations of the University of Surrey's Ethics Committee with written informed consent from all subjects. All subjects gave written informed consent in accordance with the Declaration of Helsinki. The protocol was approved by the University of Surrey's Ethics Committee.

## Author Contributions

The study was conceived of and designed by SG, JO, and HH, who also all contributed to the development of the interview schedule. Recruitment was undertaken by SG and HH. SG arranged and conducted the interviews, transcribed the recorded interviews, and completed the initial coding of the transcripts. Higher-order analysis and theme development was undertaken by SG, JO, and HH. The first manuscript draft was produced by SG. JO and HH provided comments and revisions though several iterations of the manuscript.

### Conflict of Interest Statement

The authors declare that the research was conducted in the absence of any commercial or financial relationships that could be construed as a potential conflict of interest.

## Footnotes

^1^Veterinary Medicines Directorate

^2^Animal and Plant Health Agency

^3^Agriculture and Horticulture Development Board

^4^Department for Environment, Food, and Rural Affairs
