# Supplementary material for: Shared Goals, Different Barriers: A Qualitative Study of UK Veterinarians' and Farmers' Beliefs About Antimicrobial Resistance and Stewardship
Source: Front Vet Sci. 2019 Apr 25;6:132. doi: 10.3389/fvets.2019.00132 (PMC6494936; doi:10.3389/fvets.2019.00132)
Supplement: Supplementary file 1 [file Table_1.DOCX]

Supplementary Material

# Interview Schedule for Vets and Farmers

All questions were posed to both vets and farmers, except where indicated. Vets were asked about ‘prescribing’ antimicrobials; farmers were asked about ‘using’ antimicrobials.

## Opening ‘warm-up’ questions

Ok, first I’d like to ask a little about your work experience… what livestock do you look after / keep?

- Have you always just looked after [*animals mentioned previously*]?
- [*Vets*] Do you just work with livestock, or do you ever get involved with other animals?
- [*Farmers*] Do you just keep livestock on your farm?
- [*Farmers*] How many other people work with the animals on your farm?

## Main questions about antimicrobial use

Ok, thanks. So now I’d like to talk about your role in prescribing/using antibiotics on the farms that you work with / your farm.

[*Critical Incident 1*]:

Please can you tell me about a recent example of when you had to prescribe/use antibiotics?

- Why did you decide to prescribe/use antibiotics on this occasion?
- What made you think this was the best approach to take here?
- Would you describe this case as a relatively straightforward example of when you had to decide whether to prescribe/use antibiotics?

[*Critical Incident 2*]:

[*Depending on previous answer…*] Ok, please can you tell me about another recent example of when you had to prescribe/use antibiotics, where the decision was more/less clear-cut?

- Why did you decide to prescribe/use antibiotics on this occasion?
- What made you think this what the best approach to take here?

[*Critical Incident 3*]:

Finally, please can you tell me about a recent example when you could have prescribed/used antibiotics but you decided not to?

- Why did you decide NOT to prescribe/use antibiotics on this occasion?
- What made you think this what the best approach to take here?

[*Additional questions…ask only if these topics have not come out during critical incident discussions*]

- What are your primary considerations when thinking about prescribing/using antibiotics?
- Are there any other factors that influence your decisions to prescribe/use antibiotics?
- [*Vets*] Do you ever prescribe antibiotics when it is against your clinical judgement?
  - Why / Why not?
- [*Vets - depending on previous response*] Do you ever feel under pressure to do so?
  - Why / Why not?
- [*Vets*] Do you ever consider using antibiotics off-label?
  - Why / Why not?
- [*Farmers*] Do you ever use antibiotics, but wish you didn’t have to?
  - Why / Why not?
- Do you draw upon any guidelines or protocols when you are considering whether to prescribe/use antibiotics, or what to prescribe/use?
  - If so, which ones?
- [*Vets*] Do you ever speak to farmers about the amount of antibiotics that they use?
- [*Farmers*] Do you ever speak with your vet about how you should use antibiotics?
- What types of situations might prompt you to speak to them?
- How often would you do this?
- How comfortable are you with current levels of antibiotics used on the farms on which you work / on your farm?

Do you have any other comments about antibiotic prescribing/use, either from your own experience / on your farm, or in general?

## Main questions about antimicrobial resistance (AMR) beliefs

[*Speak the word resistance throughout, don’t use abbreviation*]

Ok, so now I’d like to ask you about antimicrobial resistance as I’m interested in your thoughts and opinions on this topic.

- What does the term antimicrobial resistance mean to you?
  - Does the issue of AMR influence your management of livestock?
  - Do you think there are any consequences from AMR?
  - Are you concerned at all about AMR?
- Where do you get your information about AMR from?
  - E.g. Media? Peers? Professional media? Journals?

I’d like to ask you some questions now about AMR, that will relate firstly to animals, and then to humans

- Do you think that rates of antimicrobial resistance amongst animals are increasing, decreasing or stable?
  - Why do you think this might be?
- And do you think that rates of antimicrobial resistance amongst humans are increasing, decreasing or stable?
  - Why do you think this might be?
- Do you think antibiotic use in farming has any role to play in driving resistance, firstly, in animals?
  - And do you think antimicrobial resistance in animals is related to human health in any way?
    - [*Prompt if simple Yes/No answer*] – Please can you explain why you think that?
- Do you think antibiotic use in farming has any role to play in driving resistance in humans?
  - And do you think antimicrobial resistance in humans is related to animal welfare or food security in any way?
    - [*Prompt if simple Yes/No answer*] – Please can you explain why you think that?
- Do you think there is any exchange between animal and human bacteria of resistance?
  - [*Prompt if simple Yes/No answer*] – Please can you explain why you think that?
- What role, if any, do you think vets/farmers [vets were asked about vets first / farmers were asked about farmers first] have to play in addressing the issue of AMR?
  - If so, how much influence do you think vets/farmers have over rates of AMR?
- What role, if any, do you think farmers/vets have to play in addressing the issue of AMR?
  - If so, how much influence do you think farmers/vets have over rates of AMR?

Ok, thank you

- On a scale of 1–5, where 1 is ‘not at all’, and 5 is ‘very’, how concerned would you say you are about AMR in animals?
- And, on a scale of 1–5, where 1 is ‘not at all’, and 5 is ‘very’, how concerned would you say you are about AMR in humans?

Do you have any other comments about antimicrobial resistance?

## Suggestions for the future

You may be aware that in 2013, the UK government launched a 5-year strategy to try and slow the rates of AMR. This strategy was supported by the Chief Medical and Chief Veterinary Officers. So, given this drive from government:

- What practical initiatives might help you or your colleagues reduce/optimize the amount of antibiotics you prescribe/use on farms?
- Is there anything you would like to do differently if you could?
- Is there anything you would like vets/farmers [*participants were asked here about the other profession*] to do differently if they could?

## Closing remarks

Thank you for your time. Are there any additional comments you would like to add on the topic of prescribing or AMR?
